# Feverishness in Children

**Published:** 1905-01-21

**Authors:** 


### FEVERISHNESS IN CHILDREN.

In the January number of the *British Journal of Children's Diseases* Dr. C. W. Chapman protests against the common habit of attributing fever and other symptoms of illness in children to "the teeth." Dentition is a normal process which, if a child is healthy, need not cause any alarm, and which ought to be regarded as the cause of indisposition in a particular case only after every other possible cause has been eliminated. Contemporaneously with dentition, there are certain developmental changes in the nervous system, and also in the digestive organs preparatory to the change of diet which is about to take place, and therefore at this period these structures are particularly sensitive to all forms of irritation, and fever, convulsions, sickness, and diarrhœa are frequent occurrences in infants who are "cutting their teeth." It is surprising how the temperature will run up simply from the irritation caused by some undigested milk curds, or other hard, insoluble substance in the stomach. Besides the taking of improper food, insufficient protection from cold of the abdomen and legs is a not uncommon cause of digestive disturbances giving rise to fever in young children. Another frequent cause and one which is often overlooked, is tonsillitis. The throat should be examined in all cases of feverishness. By so doing the presence of one of the exanthemata may be detected which otherwise might elude discovery. The recognition of tonsillitis is especially valuable as indicating a rheumatic tendency. Many cases of organic heart-disease have their rheumatic history limited to attacks of tonsillitis, and a recognition of this is of considerable importance from the point of view of prophylaxis. Broncho-pneumonia may easily be overlooked in the course of ordinary examination. To avoid this error, auscultation must be performed thoroughly over every inch of the lungs or a small patch of pneumonia may be missed. The treatment of fever in children will depend entirely upon the cause; when this is an error of diet a mercurial aperient should be given, and the child should be kept on bland food for the next few days. Convalescence may be assisted by giving a few grains of sodium bicarbonate with rhubarb if the tongue has not cleaned, or two or three grains of carbonate of bismuth when gastric irritability and redness of the tongue are present.

